# Prognostic Nomogram Based on the Metastatic Lymph Node Ratio for T_1-4_N_0-1_M_0_ Pancreatic Neuroendocrine Tumors After Surgery

**DOI:** 10.3389/fonc.2022.899759

**Published:** 2022-04-27

**Authors:** Jingxiang Shi, Sifan Liu, Jisen Cao, Shigang Shan, Chaoyi Ren, Jinjuan Zhang, Yijun Wang

**Affiliations:** ^1^Department of Hepatobiliary Surgery, The Third Central Hospital of Tianjin, Tianjin, China; ^2^Tianjin Key Laboratory of Extracorporeal Life Support for Critical Diseases, The Third Central Hospital of Tianjin, Tianjin, China; ^3^Artificial Cell Engineering Technology Research Center, The Third Central Hospital of Tianjin, Tianjin, China; ^4^Tianjin Institute of Hepatobiliary Disease, The Third Central Hospital of Tianjin, Tianjin, China; ^5^School of Statistics, Tianjin University of Finance and Economics, Tianjin, China

**Keywords:** lymph node ratio, pancreatic neuroendocrine tumors, nomogram, overall survival, cancer-specific survival

## Abstract

**Purpose:**

This study aimed to investigate the prognostic significance of the metastatic lymph node ratio (LNR) in patients with pancreatic neuroendocrine tumors (pNETs) and to develop and validate nomograms to predict 5-, 7-, and 10-year overall survival (OS) and cancer-specific survival (CSS) rates for pNETs after surgical resection.

**Methods:**

The demographics and clinicopathological information of T_1-4_N_0-1_M_0_ pNET patients between 2004 and 2018 were extracted from the Surveillance, Epidemiology and End Results database. X-tile software was used to determine the best cutoff value for the LNR. Patients were randomly divided into the training and the validation groups. A Cox regression model was used in the training group to obtain independent prognostic factors to develop nomograms for predicting OS and CSS. The concordance index (C-index), calibration curves, area under the receiver operating characteristic curve (AUC) and decision curve analysis (DCA) were used to assess the nomograms. Patients were divided into four groups according to the model scores, and their survival curves were generated by the Kaplan–Meier method.

**Results:**

A total of 806 patients were included in this study. The best cutoff value for the LNR was 0.16. The LNR was negatively correlated with both OS and CSS. Age, sex, marital status, primary site, grade, the LNR and radiotherapy were used to construct OS and CSS nomograms. In the training group, the C-index was 0.771 for OS and 0.778 for CSS. In the validation group, the C-index was 0.737 for OS and 0.727 for CSS. The calibration curves and AUC also indicated their good predictability. DCA demonstrated that the nomograms displayed better performance than the American Joint Committee on Cancer (AJCC) TNM staging system (8th edition). Risk stratification indicated that patients with higher risk had a worse prognosis.

**Conclusions:**

The LNR is an independent negative prognostic factor for pNETs. The nomograms we built can accurately predict long-term survival for pNETs after surgery.

## Introduction

Pancreatic neuroendocrine tumors (pNETs) are relatively rare tumors that originate from the pancreatic neuroendocrine system (1). According to the US epidemiology survey, its incidence is less than 1 in 100,000 people, representing nearly 10% of all pancreatic tumors (2, 3). Owing to the advancement of imaging and endoscopic techniques, the detection rate of pNETs has gradually increased in recent years (4). PNETs are classified as functional and nonfunctional types based on whether they secrete hormones associated with a clinical syndrome, while nonfunctional pNETs account for most of them (4, 5). One of the characteristics of pNETs is heterogeneity. Generally, they exhibit indolent clinical features; however, they may become invasive and transform rapidly in some circumstances (6).

Surgical resection is an effective treatment for pNETs without metastases (7). One SEER database study demonstrated that surgery might greatly improve survival compared with nonsurgery interventions (114 months vs. 35 months) (8). Other studies reported that the 5-year survival rate after surgical resection of pNETs was approximately 80% (6, 9). However, due to the rarity of this disease, studies on prediction models for pNETs after surgery are deficient.

Recently, the metastatic lymph node ratio (LNR), defined as the proportion of positive nodes to total examined nodes, has been an important prognostic factor for many tumors. You et al. (10) pointed out that the LNR might predict the prognosis of patients with pancreatic cancer. Zhang et al. (11) demonstrated that the LNR was a strong negative prognostic factor for patients with colorectal cancer. Two other studies also indicated that it was negatively related to the survival of patients with gastric and small intestinal neuroendocrine tumors (12, 13). However, there are no reports of nomograms of long-term survival after resection of pNETs that incorporate LNR data.

Therefore, based on the Surveillance, Epidemiology, and End Results (SEER) database, the present study attempted to explore the correlation between the LNR and the prognosis of pNET patients and to construct nomograms to predict 5-, 7-, and 10-year overall survival (OS) and cancer-specific survival (CSS) rates for pNETs after surgery.

## Materials and Methods

### Data Collection

The SEER database is an authoritative cancer statistics database in the United States, covering approximately 30% of the US population (9). Data were extracted from the SEER database with SEER*Stat Software (version 8.3.9.2), and the Incidence SEER Research Plus Data, 9 Registries, Nov 2020 Sub (1975-2018) dataset was selected for analysis (username for login: 15881-Nov2020). Patients diagnosed with pNETs from 2004 to 2018 were identified retrospectively. The corresponding selection formula in the software was as follows: Site and Morphology, “Site recode ICD-O-3/WHO 2008” (Pancreas) and “ICD-O-3 Hist/behav” (8013/3, 8150/3-8156/3, 8240/2, 8240/3-8246/3, 8246/2, 8249/3). The following variables were extracted from the database: patient ID, age, sex, race, marital status, year of diagnosis, primary site, histologic type, grade, diagnostic confirmation, tumor size, T stage, N stage, M stage, American Joint Committee on Cancer (AJCC) TNM staging system, surgery at the primary site, the scope of regional lymph node surgery, radiation recode, chemotherapy recode, regional nodes examined, regional nodes positive, survival months, vital status recode, SEER cause-specific death classification, first malignant primary indicator and sequence number.

### Data Processing

Patients conforming to any of the following criteria were excluded: 1) surgery was not performed; 2) no regional nodes examined or the number of regional lymph nodes removed was unknown; 3) the AJCC TNM staging system belonged to M1; 4) not the first malignant tumor or multiple primary tumors; and 5) missing or unknown clinical information.

Age was regarded as a continuous variable, and other factors were treated as categorical variables. Patients who were widowed, divorced, single or separated were considered unmarried. (C25.4, islets of Langerhans), (C25.7, other specified parts of the pancreas), (C25.8, overlapping lesion of the pancreas) and (C25.9, pancreas, NOS) in the primary site were considered other. The staging system was adjusted according to the 8th edition AJCC. OS was defined as survival time until death by any cause or last follow-up, and CSS was defined as survival time until death due to pNETs (9).

### Construction and Validation of the Nomograms

The overall patients were randomly divided into a training cohort and a validation cohort at a ratio of 7:3. For the training group, univariate and multivariate Cox regression analyses were used to screen out significant variables. After that, all the independent prognostic factors in multivariate analysis were used to construct nomograms using the package “rms” in R software (14). The validation cohort was used to perform an external validation of the nomograms.

To assess the accuracy of the nomograms, we calculated Harrell’s concordance index (C-index) and drew calibration curves of the training and validation groups for internal and external validation (15). The area under the receiver operating characteristic curve (AUC) was also used to evaluate the predictive value (15). In addition, we used decision curve analysis (DCA) to determine the 5-, 7-, and 10-year survival rates of the two groups (16). Furthermore, DCA was applied to compare the AJCC staging system (8th edition), the LNR and the nomograms.

### Statistical Analysis

All statistical tests were performed using R statistical software (version 4.1.2, https://www.r-project.org, Vienna, Austria). Categorical variables were expressed as frequencies and percentages. Continuous variables were presented as the mean with standard deviation. The optimal cutoff value for the LNR was determined by the X-tile program (17). Survival curves were generated by the Kaplan–Meier method and analyzed by the log-rank test. Variables with *P* < 0.2 in univariate analysis were subjected to multivariate analysis. *P* < 0.1 in multivariate analysis was considered clinically significant. Pearson’s correlation was performed to detect collinearity among the variables. A correlation coefficient < 0.7 between two variables indicated no multicollinearity (18). Tolerance and variance inflation factor (VIF) were also used to evaluate multicollinearity between variables, with tolerance < 0.1 and VIF > 10 indicating multicollinearity (19). *P* < 0.05 was considered statistically significant.

## Results

### Patient Characteristics

**Figure 1:**
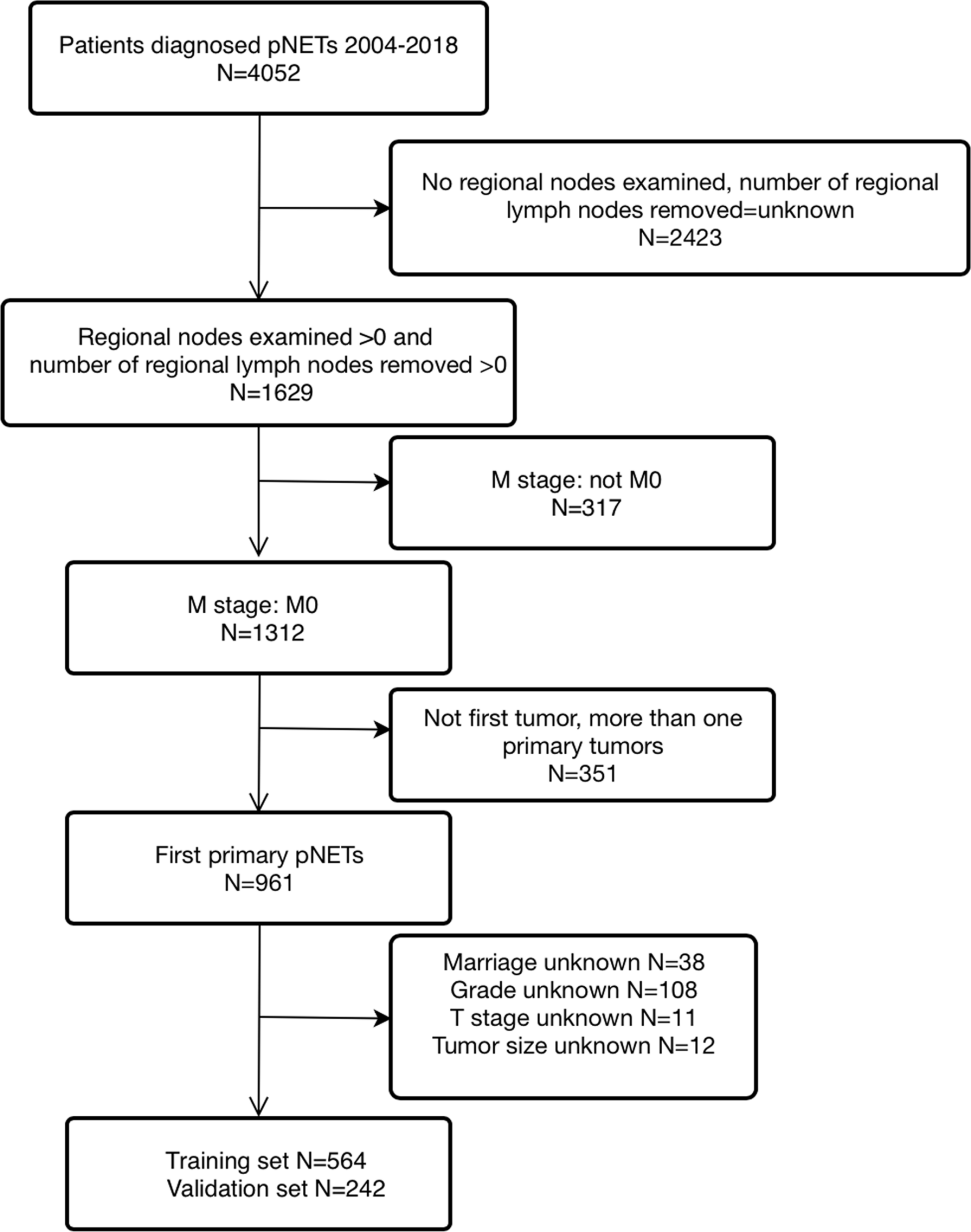
This is the flow chart of enrolled patients.

**Figure 2:**
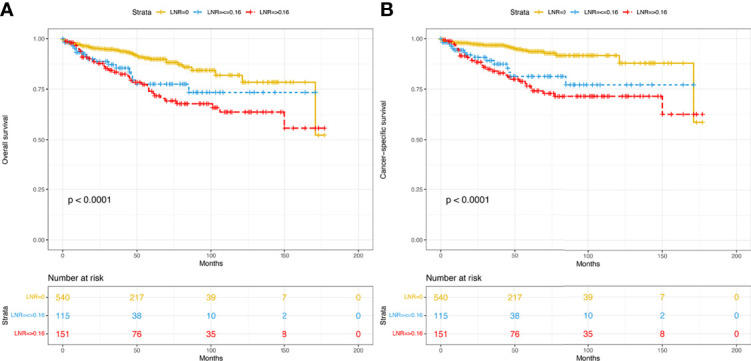
Kaplan–Meier curves of OS **(A)** and CSS **(B)** for patients with different LNRs in the overall dataset. OS, overall survival; CSS, cancer-specific survival; LNR, lymph node ratio.

A total of 806 patients with T_1-4_N_0-1_M_0_ pNETs were enrolled in this study, of which 564 cases were randomly assigned to the training group, while 242 cases were assigned to the validation group (**Figure 1**). The clinicopathological characteristics of patients in the 2 cohorts are summarized in **Table 1**. In the training group, 75 patients (13.3%) died from pNETs, and 23 (4.1%) patients died from other causes, while in the validation group, the numbers were 27 (11.2%) and 5 (2.1%), respectively. The optimal cutoff value for the LNR was 0.16. Therefore, patients were divided into three groups (LNR1: 0, LNR2: ≤ 0.16 and LNR3: > 0.16). Patients in the high LNR group had poorer OS and CSS than those in the relatively low LNR group (**Figures 2A, B****)**.

**Table 1:** Patient demographics and pathological characteristics.

Variables	Training group	Validation group
Age (year)	56.43±13.73	57.82±13.93
Sex		
Female	264 (46.8)	117 (48.3)
Male	300 (53.2)	125 (51.7)
Race		
White	413 (73.2)	183 (75.6)
Black	68 (12.1)	26 (10.7)
Others	83 (14.7)	33 (13.6)
Marriage		
Married	364 (64.5)	168 (69.4)
Unmarried	200 (35.5)	74 (30.6)
Primary site		
Head	170 (30.1)	77 (31.8)
Body	80 (14.2)	33 (13.6)
Tail	238 (42.2)	103 (42.6)
Other	76 (13.5)	29 (12)
Grade		
I	399 (70.7)	170 (70.2)
II	133 (23.6)	58 (24)
III	26 (4.6)	13 (5.4)
IV	6 (1.1)	1 (4)
Tumor size (cm)		
≤3	347 (61.5)	145 (59.9)
>3	217 (38.5)	97 (40.1)
T stage		
T1	196 (34.8)	73 (30.2)
T2	210 (37.2)	96 (39.7)
T3	142 (25.2)	69 (28.5)
T4	16 (2.8)	4 (1.7)
N stage		
N0	381 (67.6)	159 (65.7)
N1	183 (32.4)	83 (34.3)
Radiotherapy		
Yes	13 (2.3)	6 (2.5)
No	551 (97.7)	236 (97.5)
Chemotherapy		
Yes	34 (6)	15 (6.2)
No	530 (94)	227 (93.8)
Lymph nodes removed		
1 to 3	97 (17.2)	52 (21.5)
4 and more	467 (82.8)	190 (78.5)
AJCC TNM stage		
I	136 (24.1)	46 (19.0)
II	235 (41.7)	109 (45.0)
III	193 (34.2)	87 (36.0)

### Variable Screening and Nomogram Construction

**Figure 3:**
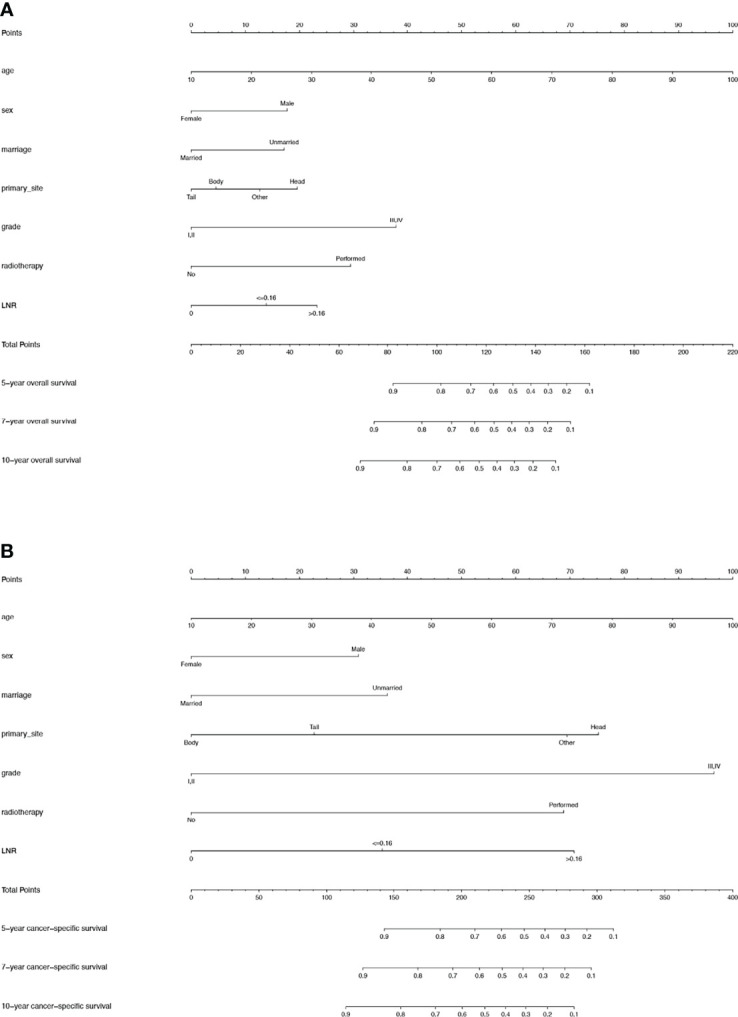
**(A)** Nomogram used to predict the 5-, 7- and 10-year OS rates of patients with T_1-4_N_0-1_M_0_ pNETs after surgery. **(B)** Nomogram used to predict the 5-, 7- and 10-year CSS rates of patients with T_1-4_N_0-1_M_0_ pNETs after surgery. OS, overall survival; pNETs, pancreatic neuroendocrine tumors; CSS, cancer-specific survival.

For the training cohort, the univariate analysis for OS revealed that age, sex, marital status, primary site, grade, tumor size, T stage, LNR, radiotherapy and chemotherapy were significantly associated with survival. Additionally, age, sex, marital status, primary site, grade, tumor size, T stage, LNR, radiotherapy, chemotherapy and lymph node dissection were significantly associated with CSS. The multivariate Cox regression analyses revealed that age, sex, marital status, primary site, grade, LNR and radiotherapy were significant for both OS and CSS (**Tables 2**, **3****)**. Therefore, these factors were included in the construction of the nomograms. Every variable was given a score in these two nomograms. Users could obtain the total score based on the individual scores of those factors and estimate the probability of survival for 5, 7 and 10 years (**Figures 3A, B**).

**Table 2:** Variables associated with overall survival (OS) according to the Cox proportional hazards regression model in the training cohort.

	Univariate analysis		Multivariate analysis	
	HR (95% CI)	*P*	HR (95% CI)	*P*
Age (years)	1.04 (1.02-1.06)	<0.001	1.04 (1.02-1.06)	<0.001
Sex				
Female	0.63 (0.40-1.01)	0.057	0.50 (0.30-0.84)	0.008
Male	Reference		Reference	
Race				
White	Reference			
Black	1.04 (0.52-2.11)	0.897		
Others	0.56 (0.24-1.31)	0.181		
Marriage				
Married	Reference		Reference	
unmarried	1.72 (1.09-2.71)	0.019	1.96 (1.19-3.23)	0.008
Primary site				
Head	Reference		Reference	
Body	0.73 (0.37-1.44)	0.360	0.75 (0.34-1.65)	0.476
Tail	0.41 (0.24-0.72)	0.002	0.52 (0.28-0.97)	0.033
Other	0.60 (0.30-1.19)	0.145	0.89 (0.42-1.88)	0.766
Grade				
I, II	Reference		Reference	
III, IV	10.05 (6.05-16.7)	<0.001	4.66 (2.35-9.22)	<0.001
Tumor size				
≤3 cm	Reference		Reference	
>3 cm	1.81 (1.14-2.85)	0.011	0.96 (0.40-2.35)	0.935
T stage				
T1	Reference		Reference	
T2	0.94 (0.50-1.75)	0.843	0.67 (0.32-1.41)	0.293
T3	2.10 (1.19-3.70)	0.010	1.50 (0.51-4.39)	0.462
T4	1.29 (0.30-5.55)	0.731	0.78 (0.17-3.62)	0.750
LNR				
0	Reference		Reference	
≤0.16	2.42 (1.29-4.54)	0.006	1.71 (0.81-3.59)	0.159
>0.16	2.67 (1.61-4.44)	<0.001	2.32 (1.36-3.93)	0.002
Radiotherapy				
Yes	9.01 (4.73-17.21)	<0.001	2.27 (0.87,5.90)	0.092
No	Reference		Reference	
Chemotherapy				
Yes	3.36 (1.88-6.01)	<0.001	1.21 (0.51-2.91)	0.669
No	Reference		Reference	
Lymph nodes removed				
1 to 3	Reference			
4 and more	1.42 (0.75-2.70)	0.281		

LNR, lymph node ratio.

**Table 3:** Variables associated with cancer-specific survival (CSS) according to the Cox proportional hazards regression model in the training cohort.

Variables	Univariate analysis		Multivariate analysis	
	HR (95% CI)	*P*	HR (95% CI)	*P*
Age (years)	1.02 (0.99-1.04)	0.106	1.02 (0.99-1.04)	0.077
Sex				
Female	0.66 (0.38-1.16)	0.152	0.58 (0.32-1.08)	0.085
Male	Reference		Reference	
Race				
White	Reference			
Black	1.20 (0.54-2.68)	0.661		
Others	0.71 (0.28-1.79)	0.463		
Marriage				
Married	Reference		Reference	
unmarried	1.95 (1.13-3.36)	0.016	2.05 (1.12-3.74)	0.020
Primary site				
Head	Reference		Reference	
Body	0.32 (0.11-0.92)	0.035	0.42 (0.13-1.34)	0.143
Tail	0.30 (0.15-0.60)	<0.001	0.45 (0.21-0.98)	0.044
Other	0.61 (0.29,1.31)	0.206	1.15 (0.49,2.68)	0.746
Grade				
I, II	Reference		Reference	
III, IV	11.71 (6.46-21.2)	<0.001	5.54 (2.41-12.72)	<0.001
Tumor size				
≤3 cm	Reference		Reference	
>3 cm	1.94 (1.12-3.37)	0.019	0.71 (0.21-2.36)	0.574
T stage				
T1	Reference		Reference	
T2	0.86 (0.38-1.91)	0.707	0.69 (0.28-1.73)	0.434
T3	2.63 (1.33-5.22)	0.006	2.12 (0.51-8.80)	0.301
T4	2.07 (0.46-9.26)	0.341	1.47 (0.30-7.23)	0.638
LNR				
0	Reference		Reference	
≤0.16	3.79 (1.76-8.16)	<0.001	1.72 (0.68-4.39)	0.252
>0.16	4.76 (2.53-8.95)	<0.001	3.24 (1.65-6.36)	<0.001
Radiotherapy				
Yes	12.2 (6.06-24.4)	<0.001	2.44 (0.90-6.64)	0.081
No	Reference		Reference	
Chemotherapy				
Yes	4.89 (2.60-9.18)	<0.001	1.46 (0.58-3.71)	0.420
No	Reference		Reference	
lymph nodes removed				
1 to 3	Reference		Reference	
4 and more	2.28 (0.91-5.74)	0.08	1.35 (0.50-3.65)	0.558

LNR, lymph node ratio.

**Figure 4:**
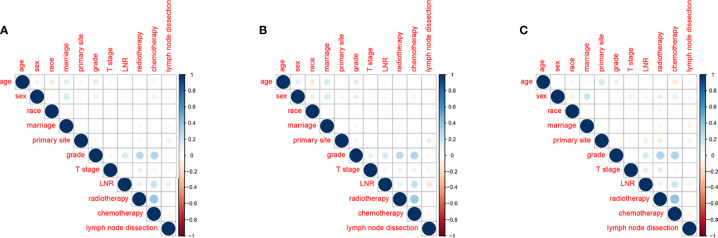
Correlations between variables in the overall dataset **(A)**, the training group **(B)** and the validation group **(C)**.

There was no significant correlation between the screened variables for the overall dataset, the training or the validation groups (**Figure 4**). Furthermore, the tolerance was >1, and the VIF was <10 for the three groups, proving no collinearity among the factors.

### Nomogram Validation

**Figure 5:**
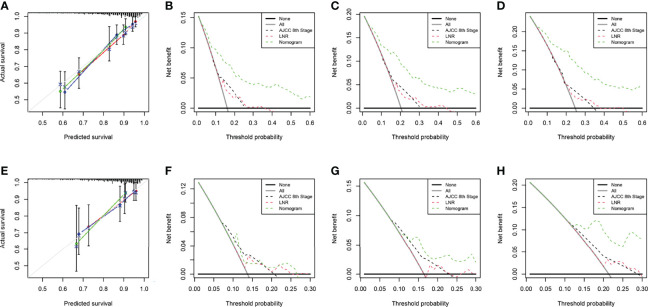
Calibration curve of the nomogram for OS prediction from the training group **(A)** and the validation group **(E)**. Decision curve analysis of the AJCC 8th edition staging system, nomogram and the LNR for the 5- **(B)**, 7- **(C)** and 10-year **(D)** OS rates of patients with T_1-4_N_0-1_M_0_ pNETs from the training group. Decision curve analysis of the AJCC 8th edition staging system, nomogram and the LNR for the 5- **(F)**, 7- **(G)** and 10-year **(H)** OS rates of patients with T_1-4_N_0-1_M_0_ pNETs from the validation group. OS, overall survival; LNR, lymph node ratio; pNETs, pancreatic neuroendocrine tumors. For calibration curves, red, blue and green lines represent 5, 7, and 10 years, respectively.

**Figure 6:**
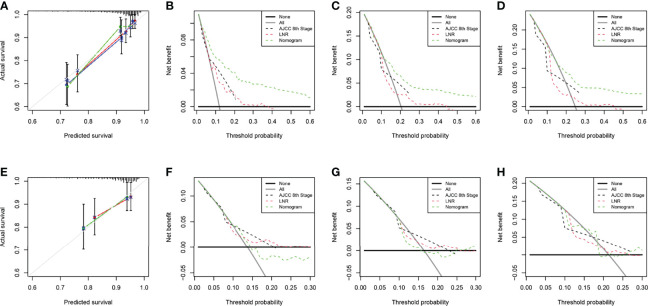
Calibration curve of the nomogram for CSS prediction from the training group **(A)** and the validation group **(E)**. Decision curve analysis of the AJCC 8th edition staging system, nomogram and the LNR for the 5- **(B)**, 7- **(C)** and 10-year **(D)** CSS rates of patients with T_1-4_N_0-1_M_0_ pNETs from the training group. Decision curve analysis of the AJCC 8th edition staging system, nomogram and the LNR for the 5- **(F)**, 7- **(G)** and 10-year **(H)** CSS rates of patients with T_1-4_N_0-1_M_0_ pNETs from the validation group. CSS, cancer-specific survival; LNR, lymph node ratio; pNETs, pancreatic neuroendocrine tumors. For calibration curves, red, blue and green lines represent 5, 7, and 10 years, respectively.

**Figure 7:**
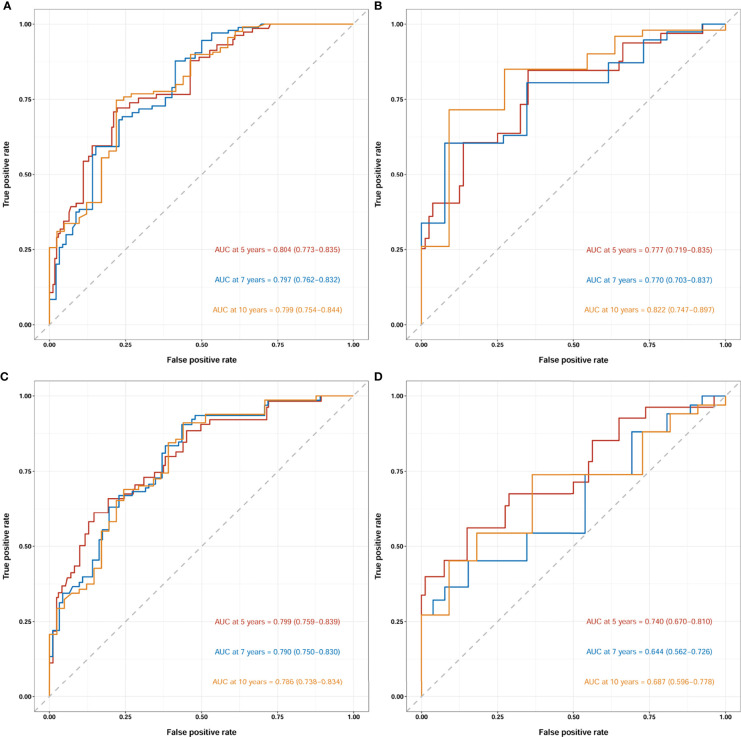
Receiver operating characteristic curves (ROCs) of the nomogram for OS prediction **(A**, training group; **B**, validation group**)** and CSS prediction **(C**, training group; **D**, validation group**)**. OS, overall survival; CSS, cancer-specific survival; AUC, area under the receiver operating characteristic curve. For ROCs, red represents 5 years, blue represents 7 years and yellow represents 10 years.

The two nomograms were validated both internally and externally. In the internal validation, the C-index was 0.771 for OS and 0.778 for CSS. In the external validation, the C-index was 0.737 for OS and 0.727 for CSS. The calibration plots were close to the standard curves for OS (**Figure 5A**) and CSS (**Figure 6A**) in the training group and for OS (**Figure 5E**) and CSS (**Figure 6E**) in the validation group. The AUC values for predicting 5-, 7-, and 10-year OS rates were 0.804, 0.797 and 0.799 in the training group (**Figure 7A**) and 0.777, 0.770 and 0.822 in the validation group, respectively (**Figure 7B**). For the 5-, 7-, and 10-year CSS rates, the AUC values were 0.799, 0.790 and 0.786 in the training group (**Figure 7C**) and 0.740, 0.644 and 0.687 in the validation group (**Figure 7D**). For both the training and validation groups, the DCA curves for OS (**Figures 5B–D, F–H**) and CSS (**Figures 6B–D, F–H**) indicated that our nomograms displayed better performance than the AJCC staging system (8th edition), and the LNR was an important factor for predicting patient prognosis.

### Risk Stratification

**Figure 8:**
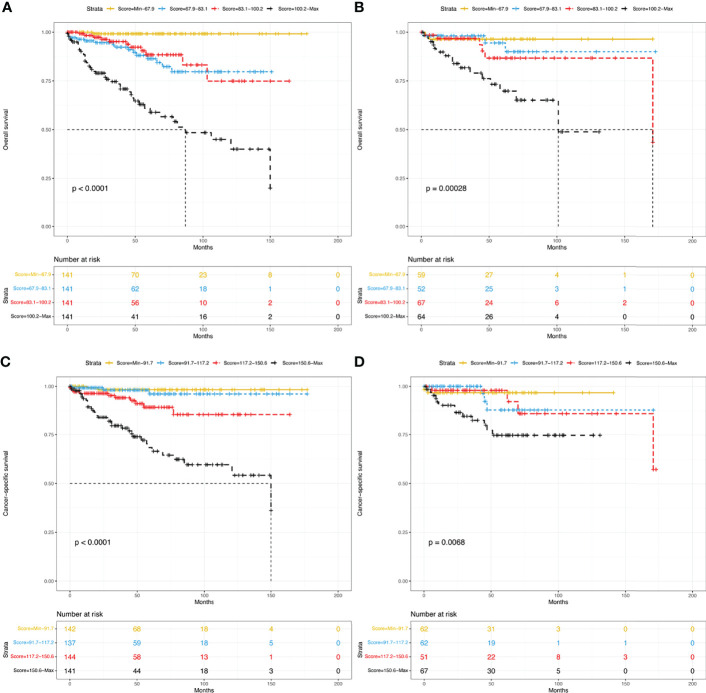
Kaplan–Meier curves of risk stratification within the training group for OS **(A)** and CSS **(C)** and within the validation group for OS **(B)** and CSS **(D)**. OS, overall survival; CSS, cancer-specific survival.

To determine the performance of the established nomograms in stratifying the risk of pNETs patients, we divided them into four groups according to the scores for OS (Min-67.9, 67.9-83.1, 83.1-100.2, 100.2-Max) and the scores for CSS (Min-91.7, 91.7-117.2, 117.2-150.6, 150.6-Max). Statistically significant differences in OS (**Figures 8A, B****)** and CSS (**Figures 8C, D****)** were found among the four groups in both the training and validation groups.

## Discussion

PNETs are heterogeneous tumors with different prognoses. Therefore, predicting the outcomes of these patients is complicated. In this study, we screened data from the SEER database and studied the factors affecting the long-term survival of T_1-4_N_0-1_M_0_ pNET patients after surgery. After that, we constructed nomograms to predict the 5-, 7-, and 10-year OS rates and CSS rates for these patients. Considering the importance of the LNR, we included it in the nomograms. The relatively high C-indices and values of AUC in the training and validation cohorts proved the good clinical predictive ability of the nomograms. The calibration plots also demonstrated their reasonable prediction probability.

The LNR has been increasingly recognized as a strong predictor of survival for many neoplasms. Teng et al. (20) noted that the LNR was a significant negative predictive variable for the OS of breast cancer patients. Tol et al. (21) reported that the LNR was an important predictor of poor survival in ampullary cancer. Using SEER data regarding pNETs, Liu et al. (22) suggested that the LNR, but not the total number of examined lymph nodes or lymph node metastasis, was an adverse prognostic factor for OS. Heidsma et al. (23) pointed out lymph node status (yes/no presence of positive node) was an independent factor to predict 5-year recurrence after resection of grade 1 and 2 nonfunctional pNETs. Ricci et al. (24) indicated that the LNR > 0.07 was associated with lower recurrence-free survival (RFS), whereas Boninsegna et al. (25) identified an LNR > 0.2 as an independent indicator of poor RFS. Another study showed that an LNR ≥ 0.5 was associated with worse CSS and that the LNR-based staging system was better than the 8th edition AJCC N staging (26). Similar to previous reports, this study showed that the LNR was a poor independent prognostic factor for both OS and CSS. Moreover, the LNR, being a ratio, eliminates the variability in lymph node sampling during surgery and is more suitable for evaluation of prognosis.

In addition to LNR, several other variables were included in the established nomograms, such as age, sex, marital status, primary site, grade and radiotherapy. Several studies have indicated that age significantly impacts the survival of pNET patients (9, 27, 28). A similar negative effect was observed in this study. There are two possible reasons for this. First, tumor resistance in elderly patients is poorer than that in young patients due to physical aging; thus, the CSS of these patients is poor (14). Second, there are far more age-related comorbid conditions in elderly patients than in young patients; therefore, the OS of elderly patients is poor. Female patients had more favorable prognoses than male patients, which was in agreement with the conclusion of Miao et al. (29). Marital status was also shown to affect both OS and CSS, consistent with previous reports (29, 30). This is because married patients usually have better psychological mentation and socioeconomic status, which may indicate a better prognosis. Similar to a previous study, the primary site of the tumor was associated with survival (29). Furthermore, histologic grade, which is an inherent characteristic of tumors, was a critical factor in predicting the prognosis of pNET patients in this study. Similar results were found in previous reports (9, 31, 32). Interestingly, this study showed that radiotherapy had some impact on survival. Iwata et al. (33) pointed out radiotherapy was an effective treatment for local disease control. However, the number of patients in that research was too small. The effect of radiotherapy on pNETs requires large sample studies.

For the first time, we developed and validated nomograms to predict 5-, 7-, and 10-year OS and CSS for T_1-4_N_0-1_M_0_ pNET patients after surgery. The nomograms included not only clinicopathological factors but also demographic factors and were more precise than the conventional staging system. Furthermore, we compared the nomograms with the AJCC TNM staging system (8th edition). DCA curves proved that our nomograms prediction ability was better than the AJCC TNM staging system. In addition, the nomograms were composed of variables that are readily available in clinical practice. With this easily used system, doctors could evaluate the risk factors for patients more precisely. Risk stratification demonstrated good applicability for patients in different stages. Therefore, patients with high risks of poor prognosis may benefit from adjuvant therapy. These may promote more specialized individualized treatment for this heterogeneous neoplasm.

There were some limitations in this study. First, it was a retrospective study based on the SEER database, and this may lead to the risk of potential selection bias. Second, several important clinicopathological factors, such as carbohydrate antigen 19-9 (CA19-9), Ki-67 index, surgical margin status, chemotherapy regimens and radiation technology, were not included due to the limitation of the SEER database, which might affect the results.

In conclusion, the present study identified that the LNR is an independent prognostic factor for pNETs. We established nomograms based on the SEER database to predict long-term survival for pNETs after surgery. These nomograms could help clinicians make tailored treatment plans for patients.

## Data Availability Statement

The datasets presented in this study can be found in online repositories. The names of the repository/repositories and accession number(s) can be found in the article/**Supplementary Material**.

## Author Contributions

JS, JZ and YW: study design and revision of the manuscript. JS: performed the literature search and wrote the manuscript. JC and SS: data collection. SL and CR: statistical analysis. JZ and YW: study supervision. All authors read and approved the final manuscript.

## Funding

This work was supported by the Tianjin Health Bureau under Grant (No. 2014KR04) and the Key Research Project of Tianjin Health and Family Planning Commission (No. 15KG114).

## Conflict of Interest

The authors declare that the research was conducted in the absence of any commercial or financial relationships that could be construed as a potential conflict of interest.

## Publisher’s Note

All claims expressed in this article are solely those of the authors and do not necessarily represent those of their affiliated organizations, or those of the publisher, the editors and the reviewers. Any product that may be evaluated in this article, or claim that may be made by its manufacturer, is not guaranteed or endorsed by the publisher.
